# Demographic, clinical, and outcome characteristics of carbapenem-resistant Enterobacteriaceae over a 10-year period (2010–2020) in Oman

**DOI:** 10.1016/j.ijregi.2022.08.001

**Published:** 2022-08-04

**Authors:** Faryal Khamis, Ibrahim Al-Zakwani, Mariya Molai, Jalila Mohsin, Samta Al Dowaiki, Maher Al Bahrani, Eskild Petersen

**Affiliations:** 1 Infectious Diseases Unit, Department of Internal Medicine, Royal Hospital, Muscat, Oman; 2 Department of Pharmacology and Clinical Pharmacy, College of Medicine and Health Sciences, Sultan Qaboos University, Muscat, Oman; 3 Department of Microbiology, Royal Hospital, Muscat, Oman; 4 Department of Anesthesia and Critical Care, Royal Hospital, Muscat, Oman; 5 Institute for Clinical Medicine, Faculty of Health Science, University of Aarthus, Denmark

**Keywords:** carbapenem resistance, bloodstream infection, Enterobacteriaceae, carbapenem-resistant Enterobacteriaceae, *Klebsiella pneumoniae*, *Klebsiella pneumoniae carbapenemase*, bacteremia, carbapenemase, carbapenemase-producing, prognostic factor, mortality, deaths, outcome

## Abstract

**Purpose:**

The incidence of carbapenem-resistant Enterobacteriaceae (CRE) has increased in the last two decades, causing significant morbidity and mortality. Our study investigated the factors associated with mortality from CRE bloodstream infection in a single center in Oman.

**Methods:**

Data from adult patients with CRE bacteremia, over a 10-year period, were retrospectively collected. Demographic and clinical characteristics were compared according to intensive care unit (ICU) admission status and mortality. A logistic regression model was used to evaluate factors associated with mortality.

**Results:**

169 cases of CRE bacteremia were identified, of whom 93 (55%) required ICU admission and 96 (56.8%) died. Patients who required ICU care were more likely to require organ transplant (15% vs 4.0%; *p* = 0.02), be on immunosuppressants (31% vs 17%; *p* = 0.035), be transferred from other hospitals (40% vs 14%; *p* < 0.001), be colonized with CRE (73% vs 43%; *p* < 0.001), have vascular lines (85% vs 42%; *p* < 0.001), be on mechanical ventilation (91% vs 9.2%; *p* < 0.001), require a longer stay (37 vs 17 days; *p* < 0.001), and have increased mortality (80% vs 29%; *p* < 0.001). In the multivariate analysis, mechanical ventilation (adjusted odds ratio (aOR) 15.3; 95% confidence interval 5.39–43.2; *p* < 0.001) and prior use of the broad-spectrum antibiotics meropenem (*p* = 0.01) and piperacillin/tazobactam (*p* = 0.026) were associated with CRE mortality.

**Conclusion:**

CRE bacteremia carries a high mortality rate in patients requiring ICU care. Implementation of infection control measures and antimicrobial stewardship programs are essential in reducing the rates of CRE BSI.

## Highlights

• CRE BSI causes significant morbidity and mortality
• Use of broad-spectrum antibiotics is a risk factor for death from CRE BSI
• ICU stay and mechanical ventilation are risk factors for death from CRE BSI
• Antibiotic use 4 days prior to blood cultures was associated with death

## Introduction

Carbapenems have been among the most effective treatment options for multidrug-resistant pathogens (Gupta et al., 2011). Active surveillance has revealed rapidly increasing incidences of carbapenem-resistant Gram-negative bacilli (CRGNB) worldwide (Gupta et al., 2011; Magiorakos et al., 2013; Guh et al., 2014; Britt et al., 2018). Nosocomial transmission of these resistant organisms poses an even greater challenge to healthcare systems due to the associated high morbidity and mortality. Carbapenem resistance has been primarily reported in *Pseudomonas* and *Acinetobacter* species (Perez et al., 2010). However, it has also emerged among species of the Enterobacteriaceae family that includes *Klebsiella pneumoniae, Escherichia coli, Enterobacter cloacae,* and *Enterobacter aerogenes* (Hussein et al., 2009). The widespread distribution of carbapenem-resistant Enterobacteriaceae (CRE) is mainly attributable to their production of carbapenemases and the plasmid-mediated horizontal transmission of the encoding genes (Cui et al, 2019). Carbapenemases can hydrolyze carbapenems and other β-lactam antibiotics, rendering them inactive. Based on the Ambler classification method (Ambler et al., 1980), they have been grouped into classes A, B, and D. Class A and class D are serine β-lactamases, while those in class B are metallo-β-lactamases (MBLs).

The prevalences of CRE and carbapenemase type tend to vary according to geographical region. In the USA, the most common carbapenemase is the class A enzyme *Klebsiella pneumoniae* carbapenemase enzyme (KPC); the class B enzyme New Delhi metallo-beta-lactamase (NDM-1) and the class D enzyme OXA-48 are predominant in Asia and the Middle East, respectively (Nordmann et al., 2011).

Several studies have shown that mortality rates are significantly higher for CRE infections than for carbapenem-susceptible Enterobacteriaceae bacteremia (CSE) (Falagas et al., 2007; Falagas et al., 2014; Amit et al., 2015; Li et al., 2017), but these studies were limited by sample size and scope. Several factors have been associated with the acquisition of infections caused by CRE; these include concomitant chronic medical conditions, the healthcare environment, and prior use of fluoroquinolones, cephalosporins, and carbapenems (Amit et al., 2015). However, the factors behind the increased death rate in cases of CRGNB bacteremia remain unclear. Identification of the risk factors would be important in foreseeing and improving the clinical outcomes.

This study evaluated the number of deaths attributable to CRE bloodstream infections and the factors associated with mortality in a tertiary care hospital in Oman.

## Methods

### Study setting

A retrospective study was conducted from 2010 to 2020 in a tertiary care hospital with 1100-bed capacity in Muscat, Oman. Demographic, microbiological, and clinical characteristics, and data on antibiotic use and clinical outcomes for patients diagnosed with CRE bloodstream infection (BSI) were collected from medical records.

### Microbiology

Species identification and susceptibility testing were performed in the clinical microbiology laboratory using a BD BACTEC Instrumented Blood Culture System (BD, USA), with BD Phoenix antibiotic susceptibility testing providing minimum inhibitory concentrations (MICs), using automated broth microdilution methodology. Carbapenem resistance was confirmed by the disk diffusion method, the MBL E test, and GeneXpert PCR (Cephid).

### Data collection and definitions

Data collected from the medical records included demographic variables (sex and age), prior hospitalization or transfer from other hospitals, hospitalization records before intensive care unit (ICU) admission, indwelling devices, concurrent conditions, previous colonization by CRE, and sites of colonization. Empirical and definitive treatment regimens were recorded, along with clinical outcomes, including length of stay and mortality. Patients with prior infection with CRE reported on admission and repeat isolates in the same admission were excluded.

CRE were defined as *Enterobacteriaceae* showing decreased susceptibility to carbapenem (diameter for imipenem ≤ 19 mm, meropenem ≤ 19 mm, and ertapenem ≤ 18 mm, or MICs for imipenem and meropenem ≥ 4 and for ertapenem ≥ 2), in line with the updated Clinical and Laboratory Standards Institute 2021 guidelines of the respective year (Performance Standards for Antimicrobial Susceptibility Testing, 28th information supplement).

CRE BSI was defined by the presence of CRE in the bloodstream, as evidenced by positive blood cultures in cases where contamination had been excluded. BSIs were classified according to standard criteria (Horan et al., 2008). Onset of BSI was defined as the date of collection of first blood culture that produced the CRE.

Attributable mortality was defined as the difference in all-cause mortality between patients with carbapenem-resistant infections admitted to ICU and those with carbapenem-resistant infections not requiring ICU care. Empirical treatment was defined as antimicrobial treatment given before the susceptibility results. Definitive treatment was defined as antimicrobial treatment given after the susceptibility results.

An Excel program was to create a data collection tool and database. The study was approved by the Institutional Research and Ethics Committee at the Royal Hospital.

### Statistical analysis

Descriptive statistics were used to analyze the data. For categorical variables, frequencies and percentages were reported. Differences between groups were analyzed using Pearson's χ^2^ test (or Fisher's exact test for expected cells < 5). For age, mean and standard deviation were used to summarize the data, while abnormally distributed variables (e.g. length of hospital stay (LOS)) were summarized using median and interquartile range and analyzed using the Wilcoxon Mann-Whitney test. A multiple logistic regression model was used to evaluate factors associated with overall cumulative mortality (i.e. age, gender, diabetes mellitus, chronic kidney disease (CKD) requiring hemodialysis, cancer, organ transplant, CRE body colonization, immunosuppression, vascular lines, mechanical ventilation, meropenem, and tazocin). Statistical analyses were conducted using STATA version 16.1 (STATA Corporation, College Station, TX, USA).

## Results

In total, 169 bacteremia patients were included in the study. The overall mean age was 58 ± 17 years, ranging from 16 years to 99 years, and 60% (101/169) were males. The three most prevalent comorbidities recorded were CKD requiring hemodialysis (57%; 96/169), diabetes mellitus (51%; 86/169), and cancer (27%; 46/169). Sixty six per cent (111/169), 60% (101/169), and 54% (92/169) of the patients had in-hospital vascular lines, a history of CRE body colonization, and were mechanically ventilated, respectively. The number of patients colonized with CRE on admission was 74 (43.7%), while the number of patients who developed CRE body colonization after antibiotic use was 26 (15%).

As shown in Table 1, those admitted to the intensive care unit (ICU) were more likely to have had an organ transplant (15% vs 4.0%; *p* = 0.02), require in-hospital vascular lines (85% vs 42%; *p* < 0.001), be on mechanical ventilation (91% vs 9.2%; *p* < 0.001), be on immunosuppressants (31% vs 17%; *p* = 0.035), have a history of CRE body colonization (73% vs 43%; *p* < 0.001), have been transferred from other hospitals (40% vs 14%; *p* < 0.001), be associated with a longer hospital stay (37 vs 17 days; *p* < 0.001), and show increased overall mortality (80% vs 29%; *p* < 0.001).

**Table 1:** Demographic and clinical characteristics stratified by intensive care unit (ICU) admission

*Characteristic, n (%) unless specified otherwise*	*All (N = 169)*	*ICU admission*		*p-value*
		*No (n = 76)*	*Yes (n = 93)*	
Demographics				
Age, mean ± SD, years	58 ± 17	60 ± 17	56 ± 16	0.125
Male gender	101 (60%)	45 (59%)	56 (60%)	0.895
Clinical				
Diabetes mellitus	86 (51%)	36 (47%)	50 (54%)	0.408
Chronic kidney disease (CKD)*	96 (57%)	40 (53%)	56 (60%)	0.322
Organ transplant	17 (10%)	3 (4.0%)	14 (15%)	0.020
Cancer	46 (27%)	29 (38%)	17 (18%)	0.004
In-hospital				
Vascular line	111 (66%)	32 (42%)	79 (85%)	< 0.001
Mechanical ventilation	92 (54%)	7 (9.2%)	85 (91%)	< 0.001
On immunosuppressants	42 (25%)	13 (17%)	29 (31%)	0.035
Transferred from other hospital	48 (28%)	11 (14%)	37 (40%)	< 0.001
History of CRE colonization	101 (60%)	33 (43%)	68 (73%)	< 0.001
Outcome				
LOS, median (IQR), days	22 (13–45)	17 (9–27)	37 (18–64)	< 0.001
Dead	96 (57%)	22 (29%)	74 (80%)	< 0.001

SD, standard deviation; CRE, carbapenem-resistant *Enterobacteriaceae*; LOS, length of hospital stay; IQR, interquartile range

⁎ CKD requiring dialysis

The demographic and clinical characteristics stratified by mortality are presented in Table 2. Those who died were more likely to have been admitted to ICU (77% vs 26%; *p* < 0.001) and be on mechanical ventilation (76% vs 26%; *p* < 0.001). Those who had prior body colonization of CRE had a tendency towards increased mortality compared with those who did not (65% vs 53%; *p* = 0.143). However, this finding did not attain statistical significance, largely due to the study's low power (26% instead of the usual 80% or above).

**Table 2:** Demographic and clinical characteristics stratified by mortality

*Characteristic, n (%) unless specified otherwise*	*All (N = 169)*	*Mortality*		*p-value*
		*No (n = 73)*	*Yes (n = 96)*	
Demographics				
Age, mean ± SD, years	58 ± 17	57 ± 17	59 ± 16	0.512
Male gender	101 (60%)	46 (63%)	55 (57%)	0.452
Clinical				
Diabetes mellitus	86 (51%)	41 (56%)	45 (47%)	0.231
Chronic kidney disease (CKD)*	96 (57%)	44 (60%)	52 (54%)	0.427
Organ transplant	17 (10)	6 (8.2%)	11 (11%)	0.609
Cancer	46 (27%)	17 (23%)	29 (30%)	0.317
In-hospital				
Vascular line	111 (66%)	42 (58%)	69 (72%)	0.052
Mechanical ventilation	92 (54%)	19 (26%)	73 (76%)	< 0.001
On immunosuppressants	42 (25%)	14 (19%)	28 (29%)	0.137
Transferred from other hospital	48 (28%)	16 (22%)	32 (33%)	0.103
History of CRE colonization	101 (60%)	39 (53%)	62 (64%)	0.143
ICU admission	93 (55%)	19 (26%)	74 (77%)	< 0.001
Outcome				
LOS, median (IQR), days	22 (13–45)	18 (12–40)	27 (15–47)	0.077

SD, standard deviation; CRE, carbapenem-resistant *Enterobacteriaceae*; ICU, intensive care unit; LOS, length of hospital stay; IQR, interquartile range

⁎ CKD requiring dialysis

Adjusting for other factors in the multivariate logistic regression model (Table 3), the only variables that were significantly associated with increased mortality were mechanical ventilation (adjusted odds ratio (aOR) 15.3; 95% confidence interval (CI) 5.39–43.2; *p* < 0.001) and the antibiotics meropenem (aOR 4.48; 95% CI 1.42–14.1; *p* = 0.01) and piperacillin/tazobactam (aOR 2.97; 95% CI 1.14–7.72; *p* = 0.026). The combined antibiotic effect (including that of meropenem and piperacillin/tazobactam) was associated with mortality in bacteremia patients (odds ratio 2.9; 95% CI 1.2–7.0; *p* = 0.018).

**Table 3:** Factors associated with mortality in bacteremia patients, utilizing a multiple logistic regression model

*Predictor*	*aOR*	*95% CI*	*p-value*
Age	1.02	1.00–1.05	0.077
Male gender	0.50	0.22–1.15	0.103
Diabetes mellitus	0.58	0.23–1.48	0.254
Chronic kidney disease requiring hemodialysis	0.99	0.38–2.56	0.978
Cancer	2.28	0.80–6.49	0.122
Organ transplant	1.24	0.23–6.73	0.802
Body colonization of CRE	0.61	0.25–1.49	0.276
Immunosuppressed	1.41	0.43–4.63	0.569
Vascular line	0.78	0.30–2.06	0.619
Mechanical ventilation	15.3	5.39–43.2	< 0.001
Meropenem	4.48	1.42–14.1	0.01
Piperacillin/tazobactam	2.97	1.14–7.72	0.026

aOR, adjusted odds ratio; CI, confidence interval; CRE, carbapenem-resistant *Enterobacteriaceae*

The multivariate logistic model was statistically significant (*p* < 0.001), reporting a pseudo *R*^2^ of 31%, while the *C*-statistic and the Hosmer-Lemeshow *p*-value were 0.85 and 0.865, respectively, denoting an overall good model fit.

The three most prevalent empiric antibiotics were piperacillin/tazobactam (27%; 45/169), meropenem (22%; 37/169), and vancomycin (6.5%; 11/169). Colistin was prescribed in only six (3.6%) patients. Overall, 67% of the patients were on antibiotics 4 days prior to blood culture. Patients who had taken antibiotics 4 days prior to collection of blood culture (as opposed to those who had not) were associated with a longer total length of hospital stay (LOS) (27 days vs 17 days; *p* = 0.002) and increased 30-day (58% vs 25%; *p* < 0.001) and overall total (71% vs 29%; *p* < 0.001) mortality.

There were no significant differences in terms of treatment duration (10 days vs 10 days; *p* = 0.753) and median duration from positive blood culture to mortality (for those who died) (5.5 days vs 8 days; *p* = 0.342) between those who were on meropenem versus those who were not. The median time to developing CRE bacteremia from admission date was longer for those on meropenem than for those who were not (15.5 days vs 1 day; *p* < 0.001). Meropenem users were associated with overall longer LOS (40 days vs 20 days; *p* = 0.023), higher 30-day mortality (68% vs 41%; *p* = 0.004), and overall total mortality (84% vs 49%; *p* < 0.001) in comparison with those who were not on meropenem.

No significant differences were observed in treatment duration (8 days vs 10 days; *p* = 0.085), median duration after positive blood culture to mortality (for those who died) (8 days vs 6 days; *p* = 0.448), median time from admission date to developing CRE bacteremia (10 days vs 2 days; *p* = 0.071), or LOS (22 days vs 23 days; *p* = 0.775) between those who were on piperacillin/tazobactam and those who were not. However, those on piperacillin/tazobactam were associated with significantly higher 30-day mortality (67% vs 40%; *p* = 0.002) and overall total mortality (73% vs 51%; *p* = 0.009).

## Discussion

This study evaluated the risk factors and outcomes in 169 patients with CRE BSIs. In our cohort, the most reported comorbidities were CKD requiring hemodialysis, diabetes mellitus, and cancer. The majority of patients had a history of CRE body colonization, vascular lines, and mechanical ventilation.

Patients with CRE bacteremia who required ICU care were more likely to have an organ transplant, be on immunosuppressants, have been transferred from other hospitals, be colonized with CRE, require in-hospital vascular lines, require mechanical ventilation, and be associated with a longer hospital stay and increased overall mortality. Higher mortality rates were also reported in patients requiring mechanical ventilation or those with prior body colonization with CRE organisms. Furthermore, in a multivariate logistic regression, CRE bacteremia was associated with a significantly higher risk of mortality in patients on mechanical ventilation and those who received meropenem or piperacillin/tazobactam several days prior to the onset of CRE bacteremia.

The association between serious CRE infection and chronic medical conditions such as diabetes mellitus, chronic renal failure, malignancies, and transplantation is well-known. In particular, due to the exposure pressure of broad-spectrum antibiotics, one third of patients with malignancies may become colonized with CRE and subsequently develop CRE bacteremia. The presence of an indwelling device has been recognized as an additional significant risk factor for CRE invasive infections in these patients. Other factors that have been reported as independent risk factors for BSI with multidrug-resistant Enterobacteriaceae include admission to the ICU, long hospital stay, use of quinolones and cephalosporins, and a history of colonization with resistant strains (Falagas et al., 2007; Schwaber et al., 2008; Falagas et al., 2014; Amit et al., 2015; Li et al., 2017; Tran et al., 2019).

In univariate analyses, CRE body colonization has been a significant factor for ICU admission among patients with CRE bacteremia. CRE colonization among hospitalized patients varies widely, ranging from 13% to 89%, while the prevalence of CRE among non-hospitalized patients has been reported as high as 30% in some countries (Schwaber et al., 2008; Debby et al., 2012; Salomão et al., 2020). Newly detected CRE may indicate either nosocomial acquisition of resistant pathogens or expansion of pre-existing, but undetected, colonization following substantial use of antibiotics such as vancomycin, cephalosporins, and antimicrobial agents with an anti-anerobic spectrum. Prolonged hospital stay, hospital-acquired infections, and treatment with a carbapenem have been found to be independent risk factors for CRE colonization (Debby et al., 2012; Salomão et al., 2020). CRE bacteremia following colonization has been shown to be common in immunocompromised patients, with a negative predictive value for CRE colonization to develop bacteremia of 99.9% and a positive predictive value of 29.3% (Salomão et al., 2020). In our cohort, prior CRE colonization was associated with a tendency for increased risk of death from CRE bacteremia. Surveillance and early detection of CRE colonization in ICUs and optimization of infection control measures are essential in preventing transmission and serious CRE infections.

Similar to previous reports (Zarkotou et al., 2011; Viale et al., 2013 Salomão et al., 2020), the mortality rate from CRE bacteremia in our cohort reached 58.9%. Several studies, including a meta-analysis by Falgas et al., have indicated that patients with CRE are two to three times more likely to die than patients with CSE. In subgroup analyses, mortality has been consistently high in patients with BSI and with infections caused by carbapenem-resistant K. pneumoniae (Falagas et al., 2014). Although patients with CRE tend to have more severe disease and comorbid conditions, the presence of CRE remains a significant predictor of mortality after adjusting for all other variables (Daikos et al., 2009; Mouloudi et al., 2010; Ben-David et al., 2012; Bleumin et al., 2012; Daikos et al., 2012; Brizendine et al., 2015).

In our study, the main predictors of death from CRE bacteremia were ICU admission and mechanical ventilation. In multivariate analysis, the two major variables found to be associated with mortality were mechanical ventilation and the use of carbapenem and piperacillin/tazobactam. Few studies have attempted to identify the factors associated with mortality among patients with CRE bacteremia. KPC-3-Kp bacteremia and bacteremia in patients admitted to the ICU have been independently associated with mortality (Neuner et al., 2011; Hussein et al., 2013; Papadimitriou-Olivgeris et al., 2014; Li et al., 2019).

Our results indicated that mechanical ventilation was a predictor of mortality among patients with CRE bacteremia. This was also demonstrated by Shi et al. (Ko et al., 2013). Mechanically ventilated patients with bacteremias tend to have high APACHE and SOFA scores on admission, longer length of stay, multiple vascular lines, and higher colonization rates of multidrug-resistance organisms (Martin et al., 2018). All these factors potentially increase the risk of death.

Our study revealed an association between exposure to agents with anti-anerobic activity and mortality. These agents may increase CRE colonization in the lower gastrointestinal tract through the suppression of gastrointestinal anerobic flora, and increase the likelihood of CRE invasion of the bloodstream. A significant association has been shown between exposure to carbapenems and hospital-acquired CRE in some multivariate models (Marchaim et al., 2012; Swaminathan et al., 2013; Brizendine et al., 2015; Martin et al., 2018; Shi et al., 2020). Similarly, in a recent study from China that included 98 patients, adverse outcomes appeared to be more likely among patients with previous carbapenem exposure and neutropenia (Li et al., 2019). Other plausible explanations for the higher mortality among patients with serious CRE infections who are receiving antimicrobials include inappropriate or delayed administration of effective antibiotics (Patel et al., 2008; Ben-David et al., 2012; Hussein et al., 2013; Brizendine et al., 2015; Trecarichi et al., 2015). In addition, monotherapy has led to a 3.8 times increased mortality risk in patients with BSI compared with those patients receiving combination therapy (Kontopidou et al., 2014; Tumbarello et al., 2015; Sheu et al., 2019). Neither of these confounders was measured in our study. Additionally, the virulence features of the carbapenem-resistant organisms may vary among isolates with different classes of carbapenemase or among strains that belong to different clones. Furthermore, some studies might have included only clonal isolates (e.g. KPC isolates in an endemic setting), while others might have included isolates from different clones (e.g. VIM producers, which are typically polyclonal), which could have affected responses to antimicrobial therapy.

The effect of piperacillin/tazobactam on mortality could be due to the emergence of multidrug-resistant bacteria, including those with carbapenem resistance, which has been highlighted in several studies (Falagas et al., 2007; Perez et al., 2011; Wang et al., 2016). In animal models, piperacillin/tazobactam has promoted colonization of *Klebsiella pneumonia* (Donskey et al., 2006) and has appeared to be less resistant to the inoculum effect (Wu et al., 2014; Harris et al., 2018). Conversely, the association of piperacillin/tazobactam with increased mortality could be a result of cofounding by indication, whereby very sick patients tend to be given broad-spectrum antibiotics. Cofounding by indication sometimes makes medications appear to increase the adverse outcomes that they are meant to prevent (Walker et al., 1996; Bosco et al., 2010).

Our study had several strengths. First, it included only true bloodstream infections, and used data from a large cohort (*N* = 169) with the aim of identifying multiple risk factors for CRE bacteremia. Second, the measurement of severity indices was based on ICU admission and mortality, and involved analyzing clinical variables using a multiple logistic regression model to ensure objectivity and reliability. Third, this study identified CRE pathogens from actual patients and not laboratory specimens. Nevertheless, several limitations need to be considered when interpreting these findings. First, are those inherent with retrospective designs, including bias during data collection. Second, the study might have been insufficiently powered to detect other, weaker confounding factors, but with potential clinically significant effects. Third, treatment aspects were not explored in terms of treatment selection, dosage adjustments, timings, or use of combination antibiotics, which could potentially have influenced the mortality rate. Finally, other variables that have were not analyzed, including APACHE score, might have affected the strength of outcomes such as ICU admission. Nevertheless, mechanical ventilation was a strong predictor of mortality, because all patients who were transferred to ICU were on mechanical ventilation.

## Conclusions

CRE bloodstream infections have high morbidity and mortality rates. The use of broad-spectrum antibiotics, admission to the ICU, and the need for mechanical ventilation were found to be independent risk factors for CRE bloodstream infections. Therefore, antimicrobial stewardship, avoidance of invasive procedures, use of strict infection control measures, and increasing hand hygiene compliance are essential strategies for the prevention of CRE bloodstream infections. Further research to re-evaluate the mortality in CRE populations, especially among patients who receive early and effective newer antibiotics, including ceftazidime/avibactam and meropenem/vaborbactam, is required.

## Conflicts of interest

The authors declare no conflicts of interest.
